# Heat-actuated valve implementation in a point-of-care, paper-based microfluidic device for infectious disease detection

**DOI:** 10.1371/journal.pone.0344750

**Published:** 2026-04-15

**Authors:** Kevin P. Jiang, Steven Bennett, Paul Yager

**Affiliations:** Department of Bioengineering, University of Washington, Seattle, Washington, United States of America; Old Dominion University, UNITED STATES OF AMERICA

## Abstract

Paper-based microfluid devices have great potential to perform complex assays like nucleic acid amplification tests (NAATs) in point-of-care (POC) and low resource settings, but have not often succeeded because there is frequently need for affordable and simple solutions to address complex fluidics and sample delivery without risk of contamination. These issues are exacerbated when multiple sample processing procedures must be automated by valve integration in the absence of advanced supportive instrumentation or trained personnel. For NAAT application in remote or home environments, valving within a POC device must facilitate precise fluidic movement and physically separate each chemical processing step. Ideally, the valve should also be affordable, easy to manufacture, robust in varying climate environments, reliable when stored under ambient conditions, affordable, and non-reactive with chemical reagents or samples. Here, we report the design and integration of two adaptable thermally-actuated valves for application in a point-of-care, paper-based rapid analysis device. Both valves are designed to facilitate the fluidic movement and control necessary to enable reverse-transcriptase loop-mediated isothermal amplification (RT-LAMP) reactions in a paper-based device. Each valve could be used independently or alongside the other valve to control fluidic movement at specific locations along the paper fluidic pathway. The valves differ in their material composition, fabrication, and mechanisms for fluidic control, but both are affordable, durable, and simple to use. We demonstrated that each valve could be used to enable RT-LAMP reactions in a paper-based device for detecting either RNA or DNA biomarkers for respiratory infectious diseases (COVID and Flu A) from human nasal swabs.

## Introduction

Paper-based microfluidics is a rapidly growing field within analytical system development, especially for lower-cost point-of-care (POC) applications [1]. Compared to conventional microfluidics, paper-based microfluidics offer numerous benefits, such as low cost of materials, simple fabrication, and lacking the need for traditional pumps or fluidic routers, which control the direction of flow [2,3]. Paper microfluidics have been used across many industries, ranging from diagnostics to environmental safety; notably, paper-based analytics devices (PAD) have emerged as a powerful tool for diagnostics development [2,4–6]. Their affordability, enabling of complex fluid flow without the need for pumps, as well as the storage of dried reagents within paper membranes make the technology especially valuable for remote and home testing. However, their translation to commercial POC platforms has been hindered by limitations in multi-step, automated fluidic control to integrate sample processing and target detection procedures [7–9]. Here, we describe the design, fabrication, and implementation of two thermally-actuated valves that are integrated into a multi-step, multiplexed, paper-based rapid analysis tool that simultaneously detects multiple respiratory pathogens (COVID and influenza A) from a single nasal swab; this work expands upon our previously published research, in which we designed a paper-based analytical device that was enabled by a simple, thermally-actuated wax valve [10]. Both valves offer unique advantages in addressing the need for adaptable fluid control in multi-step detection systems.

Many types of diagnostic assays have been implemented in paper-based analytical devices, ranging across lateral flow tests (LFTs), electrochemical sensing, nucleic acid amplification tests, and more. The demand for automated valving solutions increases with assay complexity. Lateral flow tests are used in large-scale testing for clinical and public health applications given their low-cost, rapid readout, simple protocol, and compatibility with self-testing in decentralized settings [11,12]. LFTs leverage pre-stored chemical reagents in paper membranes to rapidly immobilize and detect target analytes with only a single user step, which allow patients to quickly self-test for in remote or home environments [11]. During the COVID-19 pandemic, LFTs for detecting SARS-CoV-2 and other respiratory diseases were distributed globally by the billions, resulting in unprecedented scale of global outreach [13–17]. However, the conventional LFT’s single-step procedure limits its assay sensitivity compared to other techniques [16,17]. Electrochemical sensing has been integrated with paper-based microfluidics to enable highly sensitive devices, but these often require multiple sample clean-up and processing steps [18,19]. The potential of these systems for both infectious disease (e.g., COVID) and chronic disease (e.g., cancer, cardiovascular disease) detection at point-of-care scale is therefore reliant on efficient fluidic control *via* valving [20–23].

Nucleic acid amplification tests (NAATs) are the current gold standard of molecular testing [24]. Complex fluidic control has been critical in the translation of NAATs to POC platforms, as they require multiple processing steps such as sample lysis, inhibitor inactivation, assay rehydration, and amplification [24,25]. Isothermal amplification techniques, notably loop-mediated isothermal amplification (LAMP), are prime candidates for low-cost diagnostics for use in POC implementation given their isothermal activity, high sensitivity, and high specificity [26–29]. LAMP, which uses four to six primers to rapidly and specifically amplify target nucleic acids, is also compatible with dried reagent storage, which makes it portable and compatible with implementation in low-resource settings [10,26,30]. Fluidic control *via* valves to separate the individual steps is necessary in the absence of complex, expensive pumps and fluidic routers. Automating valve actuation would further benefit the user by minimizing possible operator errors. To be compatible with POC paper-based diagnostic devices, valves must not only be effective at blocking and controlling fluid flow, but also be affordable, automatically actuated, compatible with assay reagents, and robust across varying storage and use climates.

While many groups have developed fluidic control solutions for a multi-step assay device integration, there has yet to be a valve approach that addresses all the needs of a point-of-care (POC) platform. Phillips *et al.* demonstrated a thermally-actuated wax-ink valve on nitrocellulose membranes that could be sequentially opened or closed in both LFT and LAMP-based devices, but this valve utilized proprietary wax and could be actuated at a relatively low temperature 41^o^C that is non-optimal for operation in warmer climate regions that could reach up to 45 °C [26,30,31]. Kim *et al.* employed a solenoid-driven pressure valve that could be activated repeatedly; however, it required a dedicated pressure driven system and would be costly to implement at POC scale [32]. Iwasaki *et al*. utilized a thermo-responsive polymer valve that could expand and shrink around a critical temperature of 32 °C and was shown to be compatible with various types of reagents [33]. However, its low operating temperature limited its transportability and functionality in warmer climates. Sesen *et al.* developed a thermally-actuated, olive-oil based valving approach in a polydimethylsiloxane (PDMS) device; while this valve is shown to be durable and robust, its implementation with a PDMS-based device, limited compatibility with paper-based analytical systems, and low operating temperature reduces its widespread availability for manufacturing and implementation in low resource communities [34]. Adedokun *et al.* developed a ball-based valve that blocked a hole at the bottom of a reagent well to prevent fluid flow; this approach is limited to manual actuation by the user across multiple assay steps [35]. While many existing valve systems present promising solutions to specific operating conditions, their inability to be adaptable to POC conditions limit their translation. Coupled with each assay’s demands for different reagents and materials, there remains a need for an adaptable valve design that could address the automated fluidics needs of multi-step POC diagnostic systems.

In previous work from this lab, we demonstrated the UbiNAAT (Ubiquitous NAAT), an integrated sample-to-result paper-based microfluidic device with a USB-powered printed circuit board (PCB) for heating, two-dimensional paper network (2DPN) for fluidic transport, and paper-based RT-LAMP assay pads for simultaneous separate detection of SARS-V-CoV-2 and influenza A [10]. The device operated on an automated script controlling heaters for lysis, valve actuation, and amplification while paired with a cell phone fluorescence reader for real-time image collection of amplification detection. To control fluidics, a “air spring” wax valve was integrated on the terminal end of the 2DPN; that valve operates by stopping air flow out of the fluidic pathway, thereby blocking fluid flow into the farthest end of the 2DPN prior to thermal valve actuation. Lafleur *et al.* previously demonstrated a thermally-actuated beeswax valve striped further upstream within the 2DPN, but it required complex manufacturing [36]. The air spring valve’s simple fabrication, downstream integration, and integration into the UbiNAAT enabled multiple critical LAMP steps (lysis, sample inhibitor inactivation, assay pad rehydration by the sample, and amplification) to be performed automatically in sequence while preserving sample integrity. As a result, we demonstrated the UbiNAAT’s ability to detect multiple pathogens from a single human nasal swab through paper-based RT-LAMP [10]. In addition to RNA targets, the UbiNAAT was also implemented in the detection of DNA biomarkers from female vaginal swabs for infectious diseases such as *Chlamydia trachomatis* (CT) and *Neisseria gonorrhoeae* (NG) [37]. The low-cost and durable air spring valve further allowed the UbiNAAT to be produced affordably.

Here, we show the development, fabrication, and mechanisms of the two thermally-actuated valves that we have separately integrated into the UbiNAAT, and that could be adapted for other paper-based detection platforms. The first, denoted herein as the “in-path” valve, is a heat-shrink valve composed of polyvinyl chloride (PVC) film that could be placed in the middle of a two-dimensional paper network to directly block fluid flow from one segment to the next. The second, the air spring valve, is a wax-based valve placed at the terminal end of a 2DPN pathway to block fluid entry into the segment of the 2DPN upstream of it. We demonstrate that both valves could be assembled using low-cost materials, reliably actuated at reasonable temperatures *via* thermal resistive heating, and enable LAMP detection of either RNA or DNA biomarkers corresponding to multiple respiratory pathogens from a single nasal swab using the UbiNAAT platform. These valves, individually or in combination, present adaptable solutions for complex fluidic systems that require multiple processing steps and are shown to enable RT-LAMP in a paper-based device. We also present the advantages and disadvantages of each valve type in device design and implementation to illustrate the various use cases where each valve could be appropriate.

## Materials and methods

### In-path valve fabrication

A film of polyvinyl chloride (PVC) (Uline, Pleasant Prairie, WI), a heat-shrink polymer, was cut on a Silhouette Cameo knife plotter machine (Silhouette America, Lindon, Utah) into strips of 5 mm x 13 mm, with an additional 2 mm slit cut into the middle of the strip (see S1 Fig). With the knife blade, the slit penetrated through the PVC sufficiently to allow air to slowly pass, but did not form a substantial opening in the film. To form an airtight seal, polycaprolactone (InstaMorph, Murray, Utah) was dissolved in toluene (Sigma Aldrich, St. Louis, Mo) at a 1% w/v ratio. Three to five μL of dissolved polycaprolactone solution was pipetted onto the 2 mm line until the line was fully covered by a circle of polycaprolactone. The valve was then dried at ambient temperature for 30 minutes before storage. Both the PCL and PVC materials were selected through operational testing to ensure that fast valve actuation (opening within a minute of heating) would be achieved using printed circuit board resistive heating.

To investigate possible negative consequences of this method of sealing the slit in this valve, leachate solution from polycaprolactone dissolution was collected by submerging a prepared in-path valve in 100 μL of TE buffer (10 mM Tris, 0.1 mM EDTA) in an Eppendorf tube (Eppendorf, Hamburg, Germany), then heating at 100 °C for 5 minutes on a heat plate (VWR, Radnor, PA). Solution from three separately prepared in-path valves were then collected *via* centrifugation at 5000 RCF for 1 minute and used to prepare template solution for rehydrating lyophilized reaction mixes.

### Air spring valve fabrication

The wax valve holder was fabricated alongside the fabrication of the UbiNAAT internal device (see device fabrication below), with a wax volume of approximately 1.1 mm^3^ (see S1 Fig). The holder was fabricated using a high-powered CO2 laser cutter (VLS3.60 CO2 laser, Universal Laser Systems, Scottsdale, AZ) by rastering (*formation of a channel in the surface through laser engraving*). Dental wax (Electron Microscopy Sciences, Hatfield, PA) was melted with a soldering iron and dropped directly into the valve holder section of the UbiNAAT internal device. Once hardened, excess wax was scraped off to the level of the PMMA surface surrounding the valve. Similar to the in-path valve, the wax used for the air spring valve was selected through operational testing to ensure that fast valve actuation (opening within a minute of heating) would be achieved using printed circuit board resistive heating.

### Reverse transcription loop-mediated isothermal amplification (RT-LAMP) assay

The RT-LAMP assay was described in our previous work [10]. Briefly, the reagents consist of WarmStart LAMP Kit (New England Biolabs (NEB), Ipswich, MA), SYTO-82 fluorescent intercalating dye (Thermo Fisher Scientific, Waltham, MA), hydroxynaphthol blue (HNB) (Millipore Sigma, Burlington, WA), trehalose (Life Sciences Advanced Technologies), dextran 500 kDa (Sigma-Aldrich, St. Louis, Mo), nuclease-free water (Thermo Fisher Scientific, Waltham, MA), and assay-specific LAMP primers (S1 Table). Six primers were used for COVID-19 and 8 primers were used for Flu A RT-LAMP assays, with all sets of primer sequences acquired from literature (Integrated DNA Technologies, Skokie, IL) (S2-S3 Tables) [38,39]. For inactivating nasal swab matrix ribonucleases (RNases), the HUDSON solution (“heating unextracted diagnostic samples to obliterate nucleases”) was adapted from the literature; it was prepared with Tris(2-carboxyethyl)phosphine hydrochloride (TCEP) (Millipore Sigma) at 2.5 mM and ethylenediaminetetraacetic acid (EDTA) (Invitrogen by Life Technologies) at 1 mM [40,41]. Purified synthetic genomic RNA for SARS-CoV-2 (Twist Biosciences, San Francisco, CA) was purchased. SARS-CoV-2 virus (heat-inactivated) (ATCC, Manassas, VA) and Flu A viruses (ATCC) were purchased, then diluted in TE buffer.

RT-LAMP reactions were performed in both PCR tubes (20 μL volume) and on Whatman quartz fiber membranes (QMA) (25 μL volume) from Cytiva (VWR, Radnor, PA) [10], as described in **S1 Table**. In-tube reactions were performed on a 96-well thermocycler (CFX Opus, Biorad) at 63^o^C, while QMA-based reactions were performed in device at 64^o^C. QMA membranes were blocked by soaking in a solution of 1% BSA (GeminiBio, Sacramento CA) and 0.1% Tween 20 (Sigma-Aldrich, St. Louis, Mo) in nuclease-free water for an hour before being drained of fluid and dried overnight in a 40^o^C oven. Once lyophilization solution (containing no target) was added to both tubes (placed in a 96-well aluminum tube holder (Qiagen)) and QMA membranes (placed in a sterile 6-well plate (Costar)) at their respective volumes, both were lyophilized following protocols described in our previous work [10,30]. In short, each apparatus was quickly frozen by insertion in liquid nitrogen for 1 minute before being placed into a fast-freeze flask (Labconco) and attached to a FreeZone 2.5 liter freeze-drying benchtop lyophilizer (Labconco, Kansas City, Mo) for overnight drying with the system temperature set at -47^o^C and the vacuum at 0.03 mbar.

### Ethics Statement for human nasal swab collection

The nasal swab collection protocol was described in our previous work [10]. In short, nasal matrix was self-collected from healthy volunteers using Rhinostic polypropylene swabs (Rhinostics, Boston, MA) following a University of Washington institutional review board (IRB) approved protocol (IRB Study 00011163) from 12 October 2020–5 July 2024. Each healthy volunteer provided informed consent for their sample through a signed consent form. Recruitment for healthy volunteers was from 12 October 2020–5 July 2024. During testing, swabs were screened for SARS-CoV-2 *via* RT-LAMP, and COVID-positive samples were discarded. Prior to testing, nasal swabs were stored at 4^o^C.

### UbiNAAT device assembly and operation

**Fig 1:**
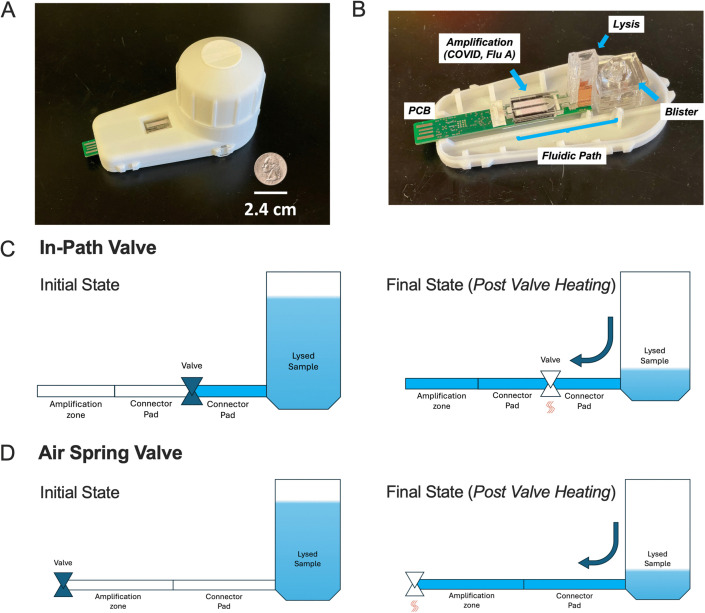
Operational schematic for in-path valve and air spring valve (A) Fully assembled UbiNAAT integrated device (B) UbiNAAT internal device schematic with labels (C) In-Path Valve Functionality. In the initial state, the in-path valve is closed and blocks fluid flow from reaching the amplification zone. Once the valve is opened *via* resistive heating, lysed sample flows past the valve and fully rehydrates amplification pads. **(D)** Air Spring Valve Functionality. In the initial state, the valve is closed, with pressure from the compressed air in the two-dimensional paper network (2DPN) restricting fluid flow out of the lysis sample chamber. Once the valve is opened *via* resistive heating, the air pressure is released and lysed sample flows out of the lysis chamber and fully rehydrates the 2DPN including the amplification pads.

The UbiNAAT device and assembly process was detailed in our previous work [10]. Briefly, the UbiNAAT consists of three major components: an RT-LAMP internal device, a custom printed circuit board (PCB), and a 3D-printed enclosure. The 3D enclosure had a marked site for nasal swab insertion. The internal device is consisted of a lysis chamber, solution blister, a 2DPN consisting of several different porous materials surrounded by a plastic housing consisting of laminated PMMA and Mylar and held together by PDMS pressure-sensitive tape (Valley Industrial Products, Huntington, NY). The 2DPN included two RT-LAMP amplification pads and one valve, in addition to connector pads. The lysis chamber was externally sheathed in with copper tape to facilitate even heating of the entire swab sample during lysis. The solution blister held 540 μL of HUDSON solution, and was activated manually *via* a pin in the 3D enclosure lid that pushed down when the lid was closed. Pin actuation on the blister pushed 300 μL of HUDSON solution into the lysis chamber to fully submerge the nasal swab. The 2D paper network was made up of 0.02% Tween-20 blocked 8950 glass fiber “connector pads” that connects the lysis chamber to the lyophilized QMA amplification pads, as shown in **Fig 1**. Air vents were added to the top PMMA layer of the internal device and covered with Tyvek membrane (DuPont, Wilmington, DE) to enable air flow. The internal device was attached to a custom printed circuit board via thermal tape (Newark Electronics, Chicago, IL) at the lysis, valve, and amplification pad regions, then enclosed by the 3D-printed enclosure.

To begin a device run, each nasal swab sample was spiked with 1.5 x 10^4^ copies of both heat-inactivated COVID virus and influenza A virus. As each QMA pad can hold no more than a 25 μL volume, there was a 12-fold dilution of the swab’s initial viral load, resulting in approximately 1250 viral copies per reaction pad. The swab was inserted into the designated opening on the 3D printed enclosure and the lid was closed, activating the push pin on the blister to push 300 μL solution into the lysis chamber. The device printed circuit board was then transported into a dark room and connected *via* USB cable to a laptop, from which TeraTerm (Tera Term Project, Japan) was used to initiate the PCB’s automated heating protocol. A cell phone fluorescence reader was fixed above the device to begin image collection at one-minute intervals over the course of an hour [10,42,43].

**Fig 2:**
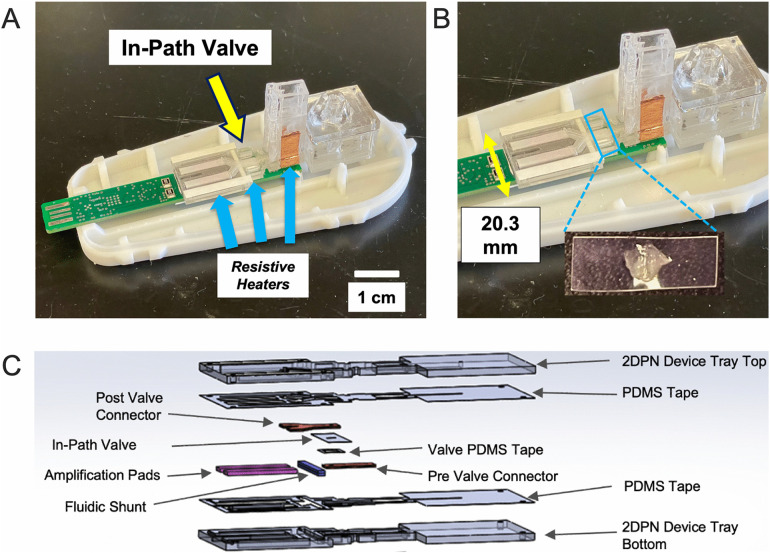
In-Path valve integration in the UbiNAAT device. **(A)** Valve location in the UbiNAAT device (arrow). **(B)** Zoomed in view showing the valve and its orientation. **(C)** Expanded view of UbiNAAT internal device schematic showing valve placement between other components.

**Fig 3:**
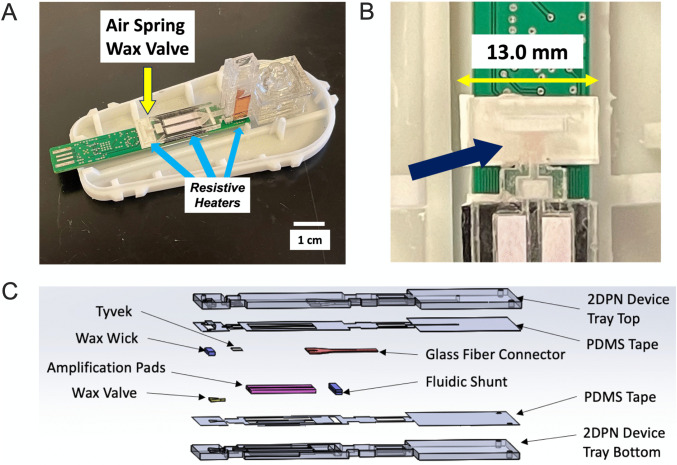
Air spring valve integration in the UbiNAAT device. **(A)** Valve location in the UbiNAAT device (arrow). **(B)** Zoomed in view showing the valve and its placement on the terminal end of the device. **(C)** Exploded view of UbiNAAT internal device schematic showing wax valve placement between other components.

Two iterations of the UbiNAAT were developed for valve testing. **Fig 2** showed the in-path valve variant of the device, with an amplification zone width of 20.3 mm. The connector pad was divided into two segments, with the in-path valve placed in between. The in-path valve was held to the bottom layer of the PMMA casing *via* PDMS tape. In **Fig 3**, the air spring valve device variant placed the valve at the terminal end of the amplification zone in the internal device. Only a single, longer connector pad was needed, and the width of the amplification zone was reduced to 13.0 mm. Additional air gaps were introduced between the amplification pads and the air spring valve segments of the internal device to reduce heat dissipation.

### Cell phone fluorescence reader, image acquisition, and data analysis

As described in previous work, a Google Nexus 5X phone was fitted with a custom black polymethyl methacrylate (PMMA) (McMaster-Carr, Elmhurst, IL) fixture that held a 12 mm wide, 12 mm focal length plano-convex lens (Edmund Optics) and two interference filters: a FES0550 emission short-pass filter (Thorlabs) and BP 587/25 excitation filter (Zeiss) [10,43]. The phone was held on a stand 8.5 cm above the amplification device. Images were taken every 60 seconds using the phone’s incandescent white balance setting, 1/5 aperture, 200 ISO, and manually focused to 8.5 cm between lens and device. Images were collected and sent *via* USB connection to a desktop computer for analysis.

The time at which significant fluorescence appeared (amplification signal liftoff time) for in-tube RT-LAMP were calculated using CFX Maestro, which applied a multivariable, nonlinear regression model to determine initial amplification. For in-pad reactions, a custom MATLAB script was used to quantify the top 1% of pixel intensity within the selected pad’s region of interest (ROI), as shown in previous works [10,44]. Average background intensity from minutes 5–10 (prior to any signal liftoff) was subtracted the ROI pixel intensity of each image. Over the course of an hour, the top 1% of the most rapidly brightening pixels in each ROI image were quantified to a fluorescence intensity that generated an amplification signal curve. Signal liftoff times were determined by steep-slope amplification signal rising above a defined fluorescence threshold of 0.02 RFU for both positive and negative samples in QMA-based RT-LAMP assays.

### Printed circuit board (PCB) heater

As previously demonstrated and validated in Jiang *et al.*, the PCB heater microprocessor (Infineon, Neubiberg, Germany) was programmed using TeraTerm to perform automated and timed heating steps [10,43]. First, lysis resistive heaters were activated at 100% power for 7 minutes, which warmed the sample to ~95^o^C for 5 minutes. The heater was then turned off for 3 minutes to allow the sample to cool to between 55^o^C and 60^o^C. The valve heater was then activated at 100% for 55 seconds, which opened the valve to allow sample to flow into the amplification pads. This setting was used for both the in-path and air spring valves. Lastly, the amplification heater temperature was maintained using a proportional-integral-derivative (PID) loop control. With a heater setpoint of either 74^o^C (for in-path valve) or 71.5^o^C (for air spring valve), each device maintained an amplification pad temperature of 64^o^C during LAMP operation. Temperature validation for the resistive heaters was previously demonstrated, with consistent heat setpoint to temperature output observed across multiple devices [10].

## Results

### Thermal validation of in-path valve

As shown in **Fig 1**, the two valve types were thermally actuated through somewhat different mechanisms. In its initial state, the in-path valve blocked the flow fluid directly upstream of the valve (**Fig 1C**). Once opened through heating, fluid flowed past the valve to rehydrate the rest of the 2DPN. The air spring valve, as seen in **Fig 1D**, blocked fluid flow in its initial state by allowing the column of fluid in the lysis chamber and the capillary suction of the fluid of the 2DPN upstream of the valve to compress air within the 2DPN until the pressures were equal. This prevented fluid flow substantially past the lysis chamber and stopped it well before the fluid could reach the amplification pads. Once the wax was heated and wicked into an adjacent membrane, the valve opened, the pressure in the amplification zones reached ambient through air flow out terminal vents, allowing fluid flow into all of the 2DPN.

**Fig 4:**
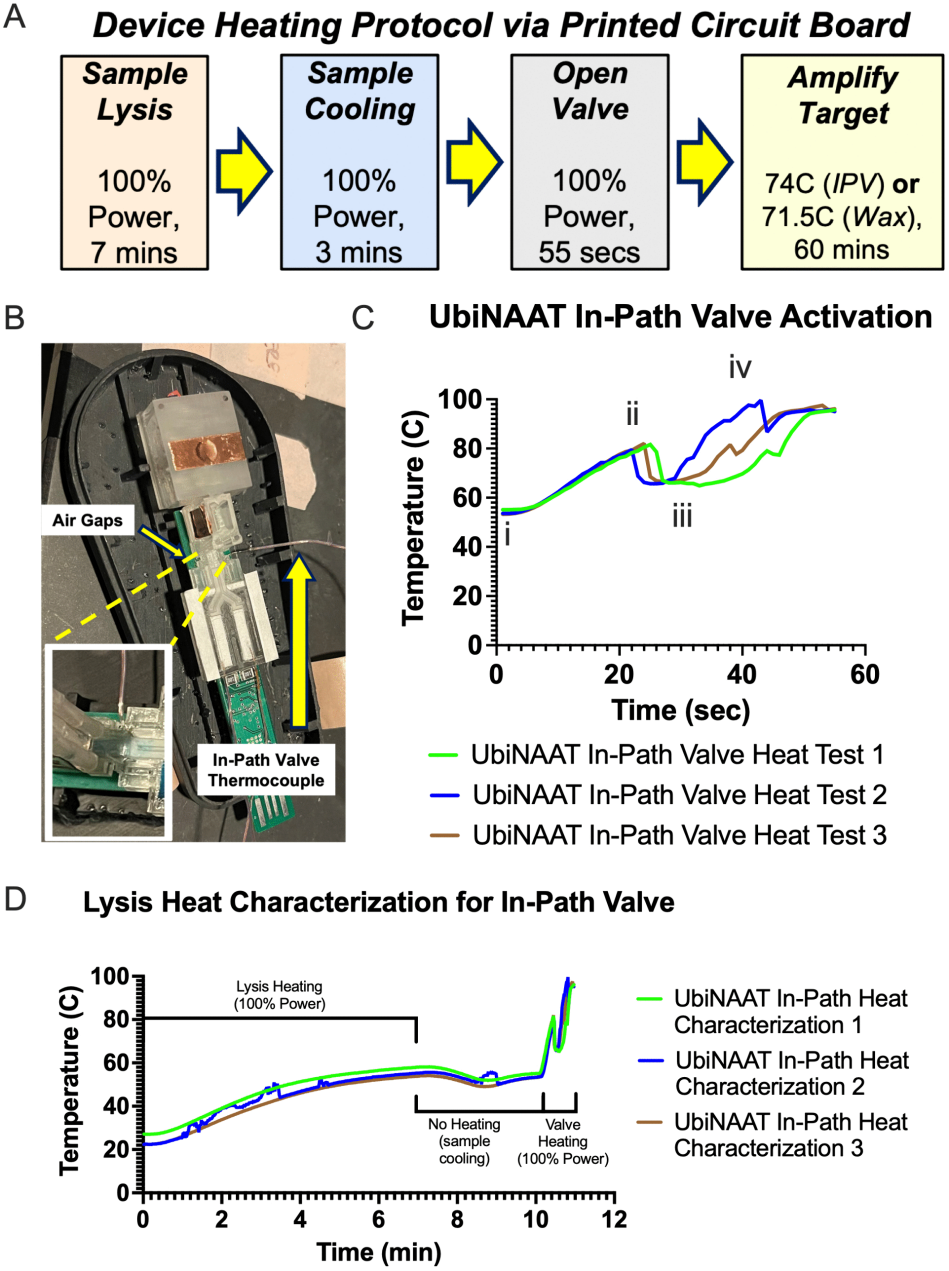
Thermal testing of valve actuation. **(A)** Schematic of the heating script as programmed on the printed circuit board (PCB) for automated lysis, cooling, valve actuation, and amplification. Parameters and duration of each step are listed across various PCB resistors. **(B)** Measurement of temperature at in-path valve during valve actuation via T-type thermocouple, which is embedded in a laser rastered channel directly beneath the valve. Zoomed-in image shown for air gaps and thermocouple at in-path valve site. **(C)** Recorded temperature at in-path valve during valve actuation. Valve actuation begins with turning on the valve resistors to 100% (i) resulting in temperature increasing until the valve opens **(ii)**, resulting in temperature falling from fluidic flow through (iii) until temperature increases following full rehydration of the two-dimensional paper network **(iv)**. The slight dip at (iv) likely indicates filling of air channels surrounding the pads post 2DPN rehydration (n = 3). **(D)** Recorded temperature at in-path valve during sample lysis (100% power for 7 minutes), sample cooling (no heating for 3 minutes), and valve activation (55 seconds) (n = 3).

We measured the actuation temperature of both the in-path valve and air spring valve to ensure that both valves would open reliably and quickly upon thermal heating. The total actuation time for both valves was set at 55 seconds, with the acceptable time-to-open being defined as less than or equal to 35 seconds. This actuation time was selected to avoid excess heating of the valve and sample above 65^o^C to avoid high-temperature solution damaging the dried assay pads upon resuspension. The remaining 20 seconds enable sample rehydration of the connector and amplification pads. **Fig 4A** illustrates the full heating protocol as programmed on the printed circuit board (PCB) for lysis, cooling, valve actuation, and amplification. Nasal matrix RNase inactivation via the HUDSON method occurred at the same temperature as lysis, as previously demonstrated in device [10].

To monitor the average time and consistency of the in-path valve actuation, we measured the valve temperature during actuation via a T-type thermocouple (Omega, Norwalk, CT) that was placed in a laser rastered channel directly below the in-path valve (see **Fig 4B**). The temperature needed to be monitored to ensure that the solution temperature entering the amplification pads did not exceed 65^o^C to avoid damaging dried LAMP reagents. The heating protocol was started and continued through the end of valve actuation, with **Fig 4C** showing valve temperature during the 55 seconds of actuation as measured by thermocouple. Across three device tests, the average in-path valve actuation time was 23.7 ± 1.2 seconds, with real-time video of in-path valve actuation shown in S2 Fig.

In addition to actuation, temperature of the valve was measured via thermocouple during sample lysis and cooling steps, as listed in **Fig 4A**. The valve temperature profile from protocol initiation through valve actuation across the three characterization tests are shown in **Fig 4D**. Starting at ambient temperature, the valve thermocouple reading reaches between 55-57^o^C following 7 minutes of lysis and stays at approximately 55^o^C during the following 3 minutes of sample cooling. Once the valve heater is activated, the valve thermocouple reading eclipses the threshold. No premature valve leakage was observed during either sample lysis or cooling steps across any of the characterization tests.

Since the polycaprolactone (PCL) on the in-path valves dissolves during actuation, its potential impact on RT-LAMP efficacy during rehydration needed to be tested. Leachate solution was collected from three separate in-path valves and used to prepare template solution along with 1000 copies of spiked SARS-CoV-2 RNA. The template solution was then used to rehydrate lyophilized reaction tubes to perform RT-LAMP on a 96-well thermocycler. Compared to the positive control (liftoff time of 13.3 mins), each PCL leachate replicate showed no significant difference in liftoff time (13.9 ± 0.4 mins) and yielded higher peak fluorescence (S3 Fig) This result indicated no significant negative impact of PCL leaching into RT-LAMP reaction mixes.

### Thermal validation of air spring valve

Valve actuation temperature for the air spring valve was evaluated *via* external heating. With a melting point of approximately 65^o^C, the wax valve actuation rate was tested on a heat plate (VWR) on top of an aluminum sheet (which represented thermal tape under the internal device). **S4 Fig** illustrates the extent of a 0.5 cm x 0.5 cm block of dental wax melting at 65^o^C, 75^o^C, and 100^o^C for 60 seconds each. While the 65^o^C and 75^o^C heating conditions still showed residual solid wax after a minute of heating, the 100^o^C heating resulted in fully melted wax after 60 seconds. The air spring valve actuates when the valve is heated at 100% power (reaching approximately 110 °C) [10]. The higher temperature used to actuate the air spring wax is less critical given its location far away from the amplification pads on the distal end of the path, ensuring that the LAMP reagents would not be damaged during valve heating.

### In-path valve integration in 2-Plex UbiNAAT operation

**Fig 5:**
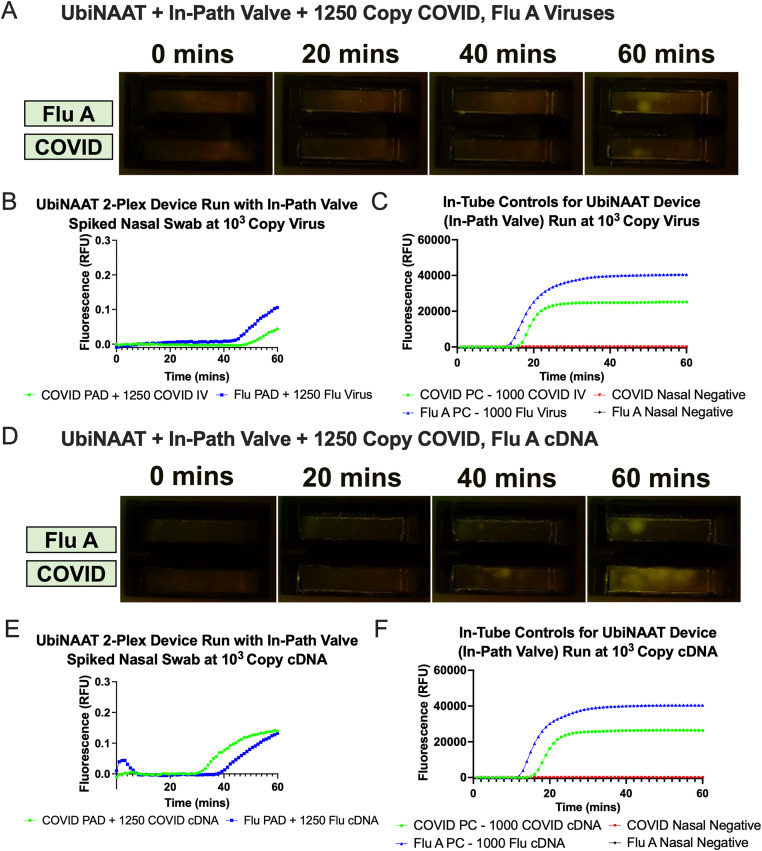
In-Path Valve integrated UbiNAAT 2-plex RT-LAMP detection of COVID inactivated virus (IV) and Flu virus spiked onto human nasal swab samples. All device and tube reactions were run with human nasal matrix. **(A)** Real-time RT-LAMP fluorescence signal of COVID and Flu A viruses over an hour. **(B)** Quantified fluorescence from 2-plex UbiNAAT to detect both COVID and Flu A viruses simultaneously. **(C)** In-tube RT-LAMP nasal controls show clear positive signals from viral samples and flat negative template control (NTC) curves (n = 1). **(D)** Real-time LAMP fluorescence signal of COVID and Flu A cDNA over an hour. **(E)** Quantified fluorescence from 2-plex UbiNAAT to detect both COVID and Flu A cDNA simultaneously. **(F)** In-tube RT-LAMP nasal controls show clear positive signals from cDNA samples and flat NTC curves (n = 1).

To demonstrate the in-path valve compatibility in a point-of-care (POC) device, we integrated it into the UbiNAAT 2-plex device to simultaneously detect SARS-CoV-2 and influenza A viruses that had both been spiked onto a single human nasal swab. To meet this goal, the valve must not only block fluid flow up to the moment of actuation, but also enable RT-LAMP detection of both SARS-CoV-2 and influenza A post-rehydration. As shown in **Fig 5**, both COVID and Flu A assay pads showed signal detection of its respective viral targets from the human nasal swab. **Fig 5A-5B** illustrates the RT-LAMP fluorescence signal and quantified results for both assays. QMA-based RT-LAMP in the UbiNAAT device showed detection at 53.7 minutes in the COVID assay pad and at 46.5 minutes in the Flu A assay pad. In-tube lyophilized RT-LAMP controls were run in parallel with spiked nasal matrix at 1000 and 500 viral copies per reaction for both assays. **Fig 5C** shows the amplification signal curves from both positive and negative nasal controls, with the COVID positive control showing amplification by 17.1 minutes (n = 1) and the Flu A positive control showing amplification by 14.2 minutes (n = 1). Neither assay’s negative nasal control showed detectable signal amplification by the end of an hour.

We further tested the in-path valve’s compatibility with UbiNAAT operations using cDNA samples for both SARS-CoV-2 and flu A. In place of a viral sample, 1.5 x 10^4^ copies of cDNA for both pathogens was spiked onto a single human nasal swab, before proceeding with UbiNAAT operations. As shown in **Fig 5D**, both COVID and Flu A assay pads showed signal detection of its respective cDNA targets from the human nasal swab. **Fig 5E** indicates that QMA-based RT-LAMP showed detection of cDNA at 41.3 minutes in the COVID assay pad and at 34.1 minutes in the Flu A assay pad. For in-tube controls, the COVID positive cDNA control at 1000 copies showed signal liftoff at 16.4 minutes (n = 1), while the Flu A positive cDNA control at 1000 copies showed signal liftoff at 12.6 minutes (n = 1). Neither assay’s negative nasal control showed detectable signal amplification by the end of an hour.

### Air spring wax valve integration in 2-Plex UbiNAAT operation

**Fig 6:**
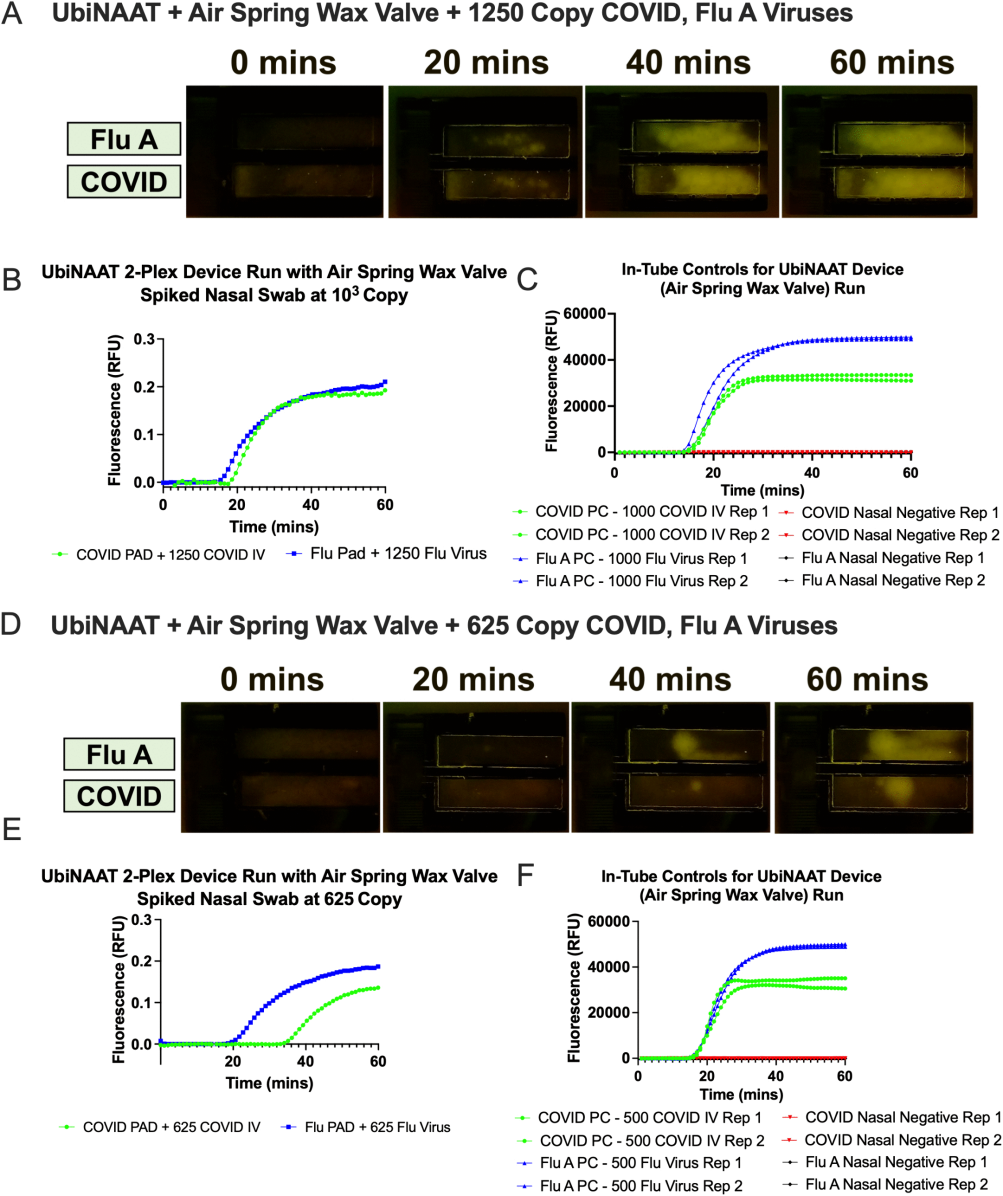
Air spring valve integrated UbiNAAT 2-plex RT-LAMP detection of COVID inactivated virus (IV) and Flu virus spiked onto human nasal swab samples. All device and tube reactions were run with human nasal matrix. **(A)** Real-time RT-LAMP fluorescence signal of COVID and Flu A viruses at 10^3^ copies per swab over an hour. **(B)** Quantified fluorescence from 2-plex UbiNAAT to detect both COVID and Flu A viruses at 10^3^ copies per swab simultaneously. **(C)** In-tube RT-LAMP nasal controls show clear positive signals from viral samples at 10^3^ copies and flat negative template control (NTC) curves (n = 2). **(D)** Real-time LAMP fluorescence signal of COVID and Flu A cDNA at 625 copies per swab over an hour. **(E)** Quantified fluorescence from 2-plex UbiNAAT to detect both COVID and Flu A viruses at 625 copies per swab simultaneously. **(F)** In-tube RT-LAMP nasal controls show clear positive signals from viral samples at 500 copies and flat NTC curves (n = 2).

The air spring wax valve’s compatibility with the UbiNAAT 2-plex device detection of SARS-CoV-2 and influenza A viruses from a single human nasal swab was also tested. Similarly to the in-path valve device tests, both pathogen viruses were spiked onto a single swab. However, two different viral loads for each pathogen were tested; the first test was performed at 1.5 x 10^4^ copies of each virus per reaction, while the second test was performed at 7500 copies of each virus per reaction. For the two tests, rehydrated QMA pads held up to 1250 and 625 copies of each virus, respectively. As shown in **Fig 6**, both COVID and Flu A assay pads showed signal detection of its respective viral targets from the human nasal swab. **Fig 6A** illustrates the amplification fluorescence signal for both assays at 1250 viral copies per reaction. **Fig 6B** shows the quantified fluorescence curves, which indicated detection at 20.6 minutes in the COVID assay pad and at 17.5 minutes in the Flu A assay pad. In-tube lyophilized RT-LAMP controls were run in parallel with spiked nasal matrix at 1000 and 500 viral copies per reaction for both assays. **Fig 6C** shows the amplification signal curves from both positive and negative nasal controls, with the COVID positive control showing amplification by 17.4 ± 0.1 minutes (n = 2) and the Flu A positive control showing amplification by 15.1 ± 0.5 minutes (n = 2). Neither assay’s negative nasal controls (n = 2) showed detectable signal amplification by the end of an hour.

When tested with 7500 viral copies of both SARS-CoV-2 and influenza A per nasal swab, the air spring wax valve integrated device also enabled early detection of each pathogen in their respective assay pad. As shown in **Fig 6D**, both COVID and Flu A assay pads showed signal detection of 625 viral copies. The quantified fluorescence curves in **Fig 6E** indicated that QMA-based RT-LAMP showed detection of cDNA at 37.2 minutes in the COVID assay pad and at 22.7 minutes in the Flu A assay pad. For in-tube controls, the COVID positive viral nasal control at 500 copies showed signal liftoff at 16.1 ± 0.3 minutes (n = 2), while the Flu A positive viral nasal control at 500 copies showed signal liftoff at 17.8 ± 0.2 minutes (n = 2). Neither assay’s negative nasal controls (n = 2) showed detectable signal amplification by the end of an hour.

## Discussion

While the two types of valves operate through different mechanisms, both valves successfully blocked fluid flow in the device prior to the completion of lysis and sample inactivation. Their differing mechanisms of actuation enable them to serve different functions. For the in-path valve, its mechanism consisted of simultaneously melting and dissolving the polycaprolactone and heat-shrinking the PVC film. The valve operating temperature of 65^o^C were selected and optimized to be compatible with the RT-LAMP operating temperature of 63^o^C, thereby reducing excessive heating of the sample prior to its flow into the assay pads. The PVC film is designed to shrink once heated above a critical temperature (~65^o^C), which in this case pull the middle slit apart to create an enlarged opening (not shown). Once the valve was open, fluid moved from one connector pad through the opening to the next, rehydrating the 2DPN. The in-path valve blocks the fluid from moving forward prior to actuation; since multiple connector pads could lead up to the same valve, multiple sample processing steps could be controlled by the same valve. While the high temperature used to actuate the valve was important for fast actuation, there was noted concern for sample stability as high heat introduced significant variability to the solution temperature as it entered the amplification zones. We hypothesize that this heat step could have caused potential damage to the nucleic acids, which may be the reason for worsened nucleic acid amplification performance. Alternatively, the air spring valve functioned by confining air within the 2DPN prior to actuation, resulting in nearly all of the fluid staying within the lysis chamber once its capillary pressure (primarily due to gravity) has equaled the pressure of the compressed air ahead in the 2DPN. Its placement on the terminal end of the 2DPN not only avoided direct and uncontrolled heating of the sample, but also prevented the valve components from interacting with the sample and RT-LAMP reagents and eliminated the possibility of contamination or wax inhibition [10]. The ability to use high temperature to actuate the air spring valve allowed for fast operation without risking sample or reagent damage. While this method is compatible with critical RT-LAMP steps (lysis, sample inactivation, and amplification), other procedures (e.g., sample concentration, filtration) will likely require additional valves along the fluidic pathway to separate the additional chemical processing steps.

The air spring valve device allowed early detection of both SARS-CoV-2 and influenza A viruses at both spike levels on the human nasal swab. By removing interaction with all reagents and membranes upstream of the amplification sites, the air spring valve eliminates possibility of contamination while still allowing for complete control of fluidic movement within the flow path. As observed in **Fig 6**, all test conditions in device showed strong fluorescence signal amplification across the entirety of the pads and early liftoff times, except at the lower copy COVID assay condition. Early positive control detection and flat negative controls indicated little to no contamination or human error (**Fig 6C**). The decrease in amplification rate for the paper-based COVID assay may have been caused by QMA blocking treatments or non-optimal storage of the lyophilized pads, as mentioned in previous studies [10,30]. With minimal interaction between the 2DPN and the isolated PMMA holder section, the air spring valve prevented additional heating of the lysed sample prior to rehydrating the amplification pads. Its design offers two distinct advantages. First, the air spring mechanism can be performed with any material that could be thermally actuated, as its lack of interaction with the rest of the 2DPN eliminates the risk of contamination or cross-reactivity in each assay for unspecific targets. Second, the air spring valve’s presence at the end of a fluid path eliminates unnecessary heating of the sample and could be easily integrated with other paper-based assay designs. Due to these advantages, we have incorporated the air spring valve into a larger sample testing of spiked human nasal swabs for greater validation of the UbiNAAT’s 2-plex detection of SARS-CoV-2 and influenza A [10]. In future work, we intend to explore integration of the air spring valve into other assay designs in addition to NAAT.

Notably, the in-path valve performed worse compared to the air spring valve when integrated into the UbiNAAT for 2-plex detection of 1.5 x 10^4^ SARS-CoV-2 and influenza A viruses from a nasal swab. As shown in **Fig 5B**, both the COVID and flu A assay pads both showed only small regions of fluorescence signal growth and signal liftoff times past 46 minutes, which is past the acceptable threshold of 30 minutes time-to-result for commercial in-vitro COVID tests [12,13]. Early signal detection for in-tube positive control across both assays and flat negative control signal curves suggested that the late amplification was not caused by assay contamination (**Fig 5C**) and is further supported by the polycaprolactone leachate not significantly impacting RT-LAMP performance (S3 Fig). When the nasal swab sample was spiked with 1.5 x 10^4^ copies of cDNA for each pathogen, amplification results significantly improved across both assays in signal liftoff time and, particularly in the COVID pad, a greater fluorescence signal across the entire pad. This suggested that the thermal actuation of the in-path valve may have negatively impacted the sample prior to its rehydration of the amplification pads. With temperature at the valve reaching up to 95^o^C, the lysed RNA sample may have been denatured, whereas DNA remained more robust to heating. The heated sample may have similarly denatured enzymes upon rehydration, which worsened the assay’s performance. Additionally, the valve heating step may have also caused increased binding of RNA to the glass fiber pad, which has been previously observed [30]. The in-path valve offered distinct advantages of being easily integrated in the middle of a 2DPN and enabling additional sample processing steps, and its actuation is compatible with DNA-based amplification system. Its compatibility with DNA amplification platforms suggests that it can readily be translated into detection assays for DNA-based pathogens, such as *Chlamydia trachomatis* (CT) and *Neisseria gonorrhoeae* (NG) [37]. Improving the in-path’s valves compatibility with RNA-based systems may be achieved by reducing the size of the valve or replacing polycaprolactone with another material to reduce the temperature required for actuation. In future iterations, we intend to refine the in-path valve’s design and explore application with other assay types.

For both valves, material composition was selected for reliable thermal actuation and robust durability in normal operating conditions. A key advantage of thermal actuation is the large availability of both automated and manual methods, including but not limited to printed circuit boards, heat plates, heat packs, and water baths. For the in-path valve, the polycaprolactone covering the PVC film slit requires higher than 60^o^C heating to dissolve, while the PVC valve itself does not open until heated above 65^o^C. Similarly, the dental wax employed in the air spring valve only consistently starts melting above 65^o^C. Stability up to at least 65^o^C indicates that either valve-device variant is capable of being transported and utilized in warm climates, with portability made even more possible by the compact form of the UbiNAAT itself. Additionally, lysis heating only raises the temperature at the in-path valve site by a consistent 30^o^C over the course of 7 minutes despite being powered at 100% (with resistive heaters reaching up to 130^o^C). This was achieved by minimizing heat transfer during lysis between the lysis chamber and in-path valve through the addition of air gaps (**Fig 4B**) and isolating device contact with the PCB heater using thermal tape. This approach allows the device to be implemented in climates reaching up to 35^o^C without concerns of premature valve leakage. In climates above 35^o^C, the device could be readily adapted by shortening the lysis heating time due to less time needed to reach lysis temperature or by swapping the valve film or wax with more robust materials. With both valves being affordable and manufacturable with simple equipment such as micropipettes and soldering iron, they can readily be produced in large scale in (and for use in) low resource settings using molding presses, automated knife plot cutters, and industrial hot air blowers.

To accommodate each valve’s design, the UbiNAAT’s internal device amplification zone dimensions needed to be adapted. With a width of 13.0 mm, the in-path valve required an amplification zone width of 20.3 mm (**Fig 2B**). The greater width caused higher heat dissipation towards the outer edges of the amplification zone, which required a higher PCB amplification setpoint of 74^o^C. The air spring valve, with a smaller width of only 3.0 mm, enabled the UbiNAAT amplification zone to be narrowed to 13.0 mm. As a result, the air spring valve device’s amplification zone is only slightly wider than the PCB heat trace, resulting in lower expected heat loss along the horizontal plane. To reduce heat loss along the length of the amplification zone, air gaps were introduced to further divide the air spring valve from the amplification pads, allowing for the use of a high valve actuation temperature (**Fig 3B**). This noticeably improved heat distribution throughout the amplification zone as noted by amplification signal uniformity, where a PCB resistive heating setpoint of 71.5^o^C enabled 64^o^C within the amplification pads. The higher amplification setpoint used for the in-path valve device may have also contributed to slower amplification, as a higher heat gradient likely caused uneven heating along the entire assay pad. This may be mitigated by reducing the size of the in-path valve and the connector pads to equalize the width of the two devices.

## Conclusions

We have demonstrated two adaptable designs for thermally-actuated valves to enable complex multi-step fluidic control, and demonstrated both in a multiplexed, paper-based rapid analysis tool that simultaneously detected either RNA (air spring valve) or DNA (in-path valve) targets for multiple respiratory pathogens (COVID and influenza A) from a nasal swab via RT-LAMP. Both valves showed reliable thermal actuation, are robust in their functionality, and are simple to manufacture. The flexibility to insert either valve in the middle or end of a paper-based system enables compatibility with different assay types depending on pathogen target, reagent, or heating compatibility. The heat durability of the materials used in both valves also encourages application in POC settings and diverse climates. We believe that these valves can be implemented across many microfluidic systems (including those without microporous materials forming the fluidic channels) to address multi-step fluidic needs. Future work from our group will report on demonstration of multi-step assay devices incorporating complex, valve-enabled sample processing of human samples for highly sensitive detection of RNA or DNA-based pathogens in a sample-to-result point-of-care platform.

## Supporting information

S1 Table Reverse transcription loop-mediated isothermal (RT-LAMP) assay reagents for tube and paper-based recipes. (DOCX)

S2 Table Nucleotide sequences of LAMP primers that target the pandemic influenza A gene. (DOCX)

S3 Table Nucleotide sequences of LAMP primers that target the SARS-CoV-2 N gene. (DOCX)

S1 Fig Valve dimensions and assembly (A) In-path valve dimensions and schematic for polyvinyl chloride film strip (above) and assembled valve post-polycaprolactone solution addition (below). (B) Air spring valve holder dimensions and schematic in UbiNAAT device (above) and post-addition of wax for valve assembly (below).
(DOCX)

S2 Fig Real-time video of in-path valve actuation. (DOCX)

S3 Fig Impact of polycaprolactone leachate on RT-LAMP efficacy. Leachate extracted from heating in-path valve shows no impact on RT-LAMP fluorescence signal compared to positive control.
(DOCX)

S4 Fig Thermal testing of dental wax used in air spring valve. 0.5 cm x 0.5 cm wax blocks were prepared at room temperature. Wax blocks were separately heated for 60 seconds at 65^o^C, 70^o^C, and 100^o^C to evaluate extent of melting.
(DOCX)

S5 Fig Real-time video of air spring valve actuation. (DOCX)
