# Supplementary material for: Heat-actuated valve implementation in a point-of-care, paper-based microfluidic device for infectious disease detection
Source: PLoS One. 2026 Apr 15;21(4):e0344750. doi: 10.1371/journal.pone.0344750 (PMC13082622; doi:10.1371/journal.pone.0344750)
Supplement: S1 Table — (DOCX) [file pone.0344750.s001.docx]

**S1 Table.** Reverse transcription loop-mediated isothermal (RT-LAMP) assay reagents for tube and paper-based recipes

| Reagent | Final Concentration (In-tube) | Final Concentration (QMA) |
| --- | --- | --- |
| WarmStart LAMP Kit | 1X | 1X |
| SYTO-82 | 7.5 µM | 7.5 µM |
| Hydroxynaphthol Blue | 40 µM | 40 µM |
| Assay specific primers (See Tables S1-S4) | varies | varies |
| Trehalose | 10% | 10% |
| Dextran, 500 kD | 0.5% | 0.5% |
| Sample | 1 µL in volume | 1 µL in volume |
| Nuclease-Free H_2_O | Fill up to 20 µL | Fill up to 25 µL |
