# Supplementary material for: Heat-actuated valve implementation in a point-of-care, paper-based microfluidic device for infectious disease detection
Source: PLoS One. 2026 Apr 15;21(4):e0344750. doi: 10.1371/journal.pone.0344750 (PMC13082622; doi:10.1371/journal.pone.0344750)
Supplement: S2 Table — (DOCX) [file pone.0344750.s002.docx]

**S2 Table.** Nucleotide sequences of LAMP primers that target the pandemic influenza A gene.

| Primer | Sequence (5’ – 3’) | Final Concentration |
| --- | --- | --- |
| F3-1 | GACTTGAAGATGTCTTTGC | 0.1 μM |
| F3-2 | GACTGGAAAGTGTCTTTGC | 0.1 μM |
| B3-1 | TRTTATTTGGGTCTCCATT | 0.1 μM |
| B3-2 | TRTTGTTTGGGTCCCCATT | 0.1 μM |
| FIP | TTAGTCAGAGGTGACARRATTGCAGATCTTGAGGCTCTC | 1.6 μM |
| BIP | TTGTKTTCACGCTCACCGTGTTTGGACAAAGCGTCTACG | 1.6 μM |
| Loop F | GTCTTGTCTTTAGCCA | 0.4 μM |
| Loop B | CMAGTGAGCGAGGACTG | 0.4 μM |
