# Supplementary material for: Heat-actuated valve implementation in a point-of-care, paper-based microfluidic device for infectious disease detection
Source: PLoS One. 2026 Apr 15;21(4):e0344750. doi: 10.1371/journal.pone.0344750 (PMC13082622; doi:10.1371/journal.pone.0344750)
Supplement: S3 Table — (DOCX) [file pone.0344750.s003.docx]

**S3 Table.** Nucleotide sequences of LAMP primers that target the SARS-CoV-2 N gene.

| Primer | Sequence (5’ – 3’) | Final Concentration |
| --- | --- | --- |
| F3 | TGGCTACTACCGAAGAGCT | 0.2 μM |
| B3 | TGCAGCATTGTTAGCAGGAT | 0.2 μM |
| FIP | TCTGGCCCAGTTCCTAGGTAGTCCAGACGAATTCGTGGTGG | 1.6 μM |
| BIP | AGACGGCATCATATGGGTTGCACGGGTGCCAATGTGATCT | 1.6 μM |
| Loop F | GGACTGAGATCTTTCATTTTACCGT | 0.4 μM |
| Loop B | ACTGAGGGAGCCTTGAATACA | 0.4 μM |
