# Supplementary material for: Heat-actuated valve implementation in a point-of-care, paper-based microfluidic device for infectious disease detection
Source: PLoS One. 2026 Apr 15;21(4):e0344750. doi: 10.1371/journal.pone.0344750 (PMC13082622; doi:10.1371/journal.pone.0344750)
Supplement: S1 Fig — (B) Air spring valve holder dimensions and schematic in UbiNAAT device (above) and post-addition of wax for valve assembly (below). (DOCX) [file pone.0344750.s004.docx]

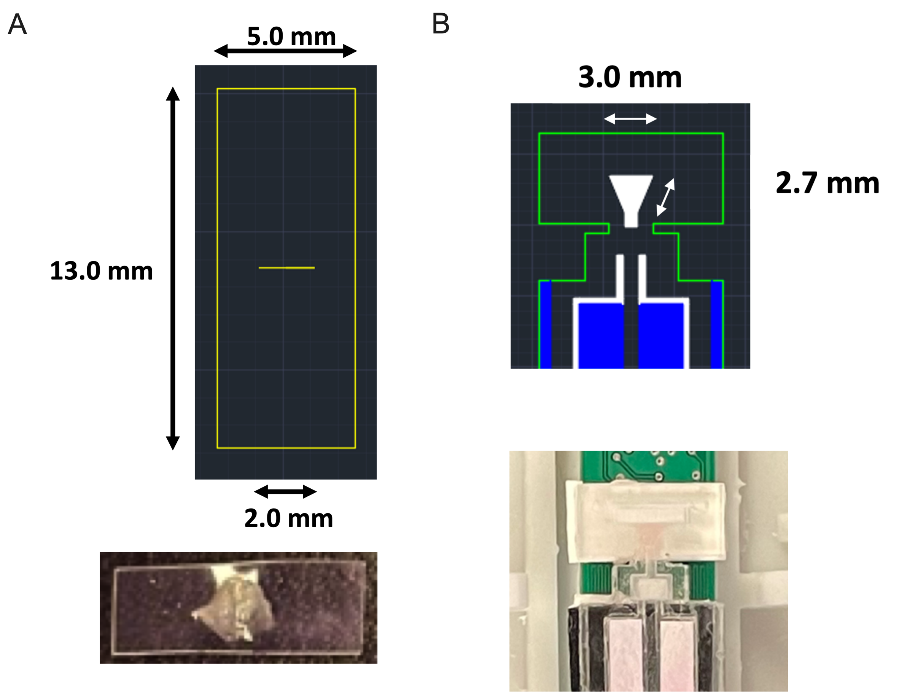


**S1 Fig.** Valve dimensions and assembly (A) In-path valve dimensions and schematic for polyvinyl chloride film strip (above) and assembled valve post-polycaprolactone solution addition (below). (B) Air spring valve holder dimensions and schematic in UbiNAAT device (above) and post-addition of wax for valve assembly (below).
