# Supplementary material for: Heat-actuated valve implementation in a point-of-care, paper-based microfluidic device for infectious disease detection
Source: PLoS One. 2026 Apr 15;21(4):e0344750. doi: 10.1371/journal.pone.0344750 (PMC13082622; doi:10.1371/journal.pone.0344750)
Supplement: S3 Fig — Leachate extracted from heating in-path valve shows no impact on RT-LAMP fluorescence signal compared to positive control. (DOCX) [file pone.0344750.s006.docx]

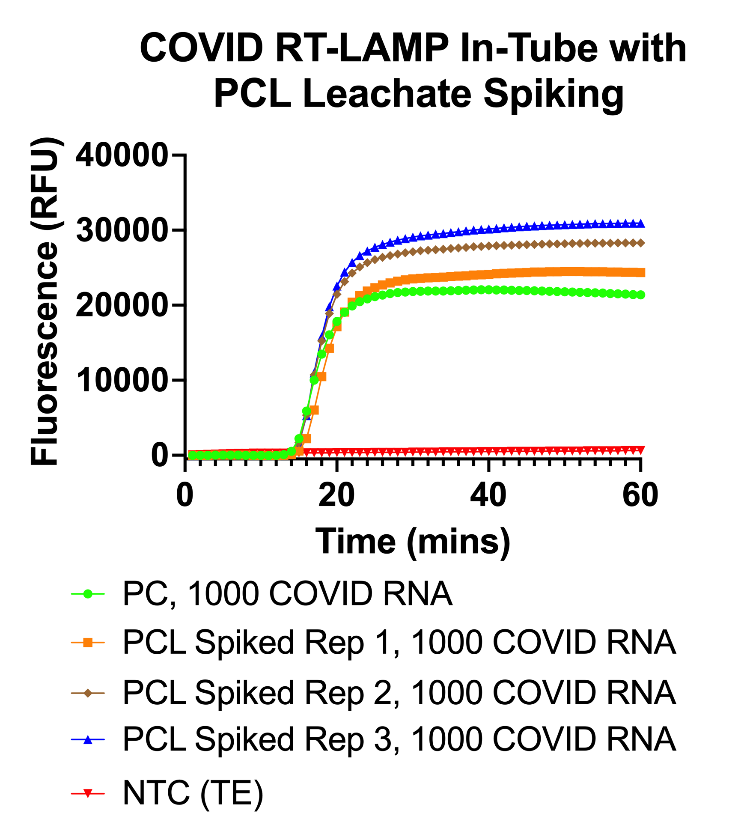


**S3 Fig.** Impact of polycaprolactone leachate on RT-LAMP efficacy. Leachate extracted from heating in-path valve shows no impact on RT-LAMP fluorescence signal compared to positive control.
