# Supplementary material for: Heat-actuated valve implementation in a point-of-care, paper-based microfluidic device for infectious disease detection
Source: PLoS One. 2026 Apr 15;21(4):e0344750. doi: 10.1371/journal.pone.0344750 (PMC13082622; doi:10.1371/journal.pone.0344750)
Supplement: S4 Fig — 0.5 cm x 0.5 cm wax blocks were prepared at room temperature. Wax blocks were separately heated for 60 seconds at 65oC, 70oC, and 100oC to evaluate extent of melting. (DOCX) [file pone.0344750.s007.docx]

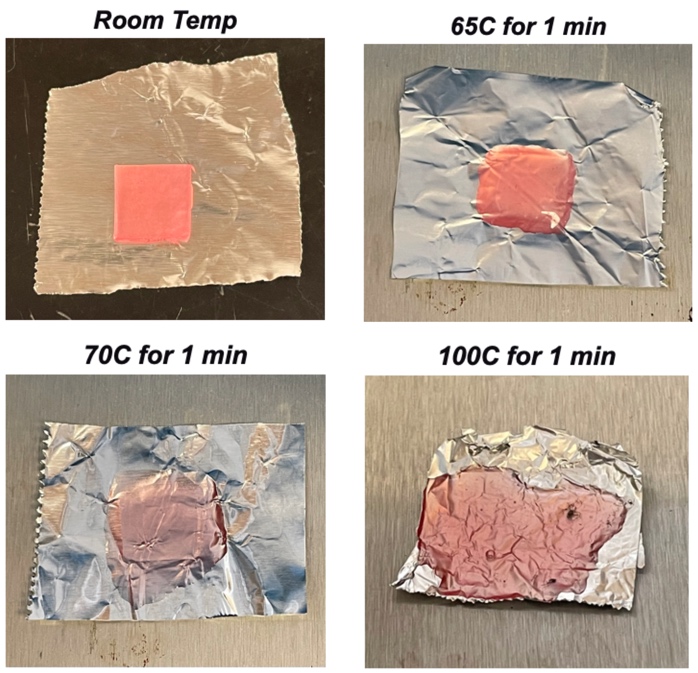


**S4 Fig.** Thermal testing of dental wax used in air spring valve. 0.5 cm x 0.5 cm wax blocks were prepared at room temperature. Wax blocks were separately heated for 60 seconds at 65^o^C, 70^o^C, and 100^o^C to evaluate extent of melting.
