# Supplementary material for: Heat-actuated valve implementation in a point-of-care, paper-based microfluidic device for infectious disease detection
Source: PLoS One. 2026 Apr 15;21(4):e0344750. doi: 10.1371/journal.pone.0344750 (PMC13082622; doi:10.1371/journal.pone.0344750)
Supplement: S5 Fig — (DOCX) [file pone.0344750.s008.docx]

[
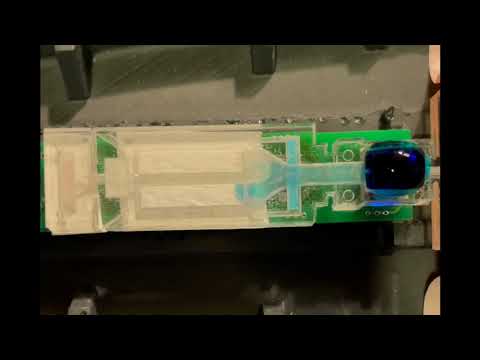
](https://www.youtube.com/embed/rPSaN6iyS3s?feature=oembed)

<https://www.youtube.com/watch?v=rPSaN6iyS3s>

**S5 Fig.** Real-time video of air spring valve actuation.
